# Royal Academy of Medicine, Paris

**Published:** 1844-04-20

**Authors:** 


### ROYAL ACADEMY OF MEDICINE, PARIS.

Among the proceedings of this body in November last, we find a report of MM. Lagneau, Gimelle, and Berard, on several surgical cases, addressed to the Academy by M. Lépine, surgeon to the Hospital of Chalons sur-Saône,

### WOUND OF THE LEFT HYPOCHONDRIUM.—ESCAPE OF THE STOMACH.—ILLUSTRATION OF THE TRUE MECHANISM OF VOMITING.

This case was one of a penetrating wound of the abdomen, followed by escape of the stomach, the transverse colon, and the epiploon, cured in twenty-one days.

A labourer, endeavouring to place a yoke on the head of a bull, received several wounds in the abdomen from his horns. One of these, about eight inches in length, occupied the left superior lateral region of the abdomen, following the margin of the false ribs and of the last true one, as far as the xiphoid appendix. The patient was able to return home on foot, a distance of about a hundred feet; on his way he in vain endeavoured to vomit. M. Lépine arrived about two hours after the accident, and found that the wound allowed the stomach, enormously distended, to escape, as also the omentum and transverse colon. The stomach appeared strangulated by the wound; some of its veins were swollen to the size of a common quill.

The first thing done by the surgeon was to return the protruded parts. The reduction of the colon was attempted and effected, although with some difficulty, owing to continued nausea. M. Lépine then applied both hands on the large curvature of the stomach, without being able, however, to circumscribe it entirely, and endeavoured, by pressure, to return a portion of the gases which distended the organ. For a long time the efforts made by the patient to vomit, efforts which were repeated at very short intervals, prevented the reduction; as soon as a portion of the stomach had been returned, the spasmodic contraction of the diaphragm and of the abdominal muscles overcame the resistance offered by the hands of the operator, and re-expelled the part. At last, by perseverance and gentle pressure, the reduction of the stomach was accomplished, and that of the omentum soon followed.

During all the time that the stomach was out of the abdomen, M. Lépine neither saw nor felt it contract, although, in order to give rise to contraction, he immersed his hands in cold water previously to placing them on it. The reduction had scarcely been accomplished when, to the nausea and vain efforts to vomit which had existed since the accident, succeeded true vomiting, which relieved the stomach of a quantity of food that the patient had taken half an hour before receiving the wound.

We have not spoken of the other less severe wounds which occupied the abdominal parietes. One of them, nevertheless, is worth noticing; the horn had torn the teguments at the level of one of the external inguinal rings, and, following the inguinal canal, penetrated to the peritoneum. The intestines were seen at the bottom of this wound, which presented also to view the spermatic cord, quite denuded.

The margins of the solution of continuity which had allowed the stomach to escape, were brought together by means of the quilled suture; and a fold of linen was placed in the wound of the inguinal region.

The results of the wound were not serious. The patient only experienced slight pains, which gave way after two bleedings; he had scarcely any fever. A slight swelling of the lips of the wound, which supervened forty-eight hours after the operation, obliged M. Lépine to loosen the sutures, which were only definitively withdrawn on the sixteenth day. The wounds were all cicatrised, and the cure was complete, on the twenty-first day. Since then (1825) unto the present time, the patient has remained perfectly well.

*Remarks.*—This interesting case not only bears directly on the mechanism of vomiting, but presents several remarkable features, to which we beg to draw the attention of the Academy:—1. The escape of the stomach from a wound of the abdomen is very rare. 2. The difficulty experienced in reducing the organ being attributable to its extreme distention by gases, was not puncture with a small trocar indicated? 3. The success of the quilled suture proves the groundlessness of the fears entertained by some surgeons, and more especially by the illustrions Larrey, whom we lost last year, with reference to the use of this suture in wounds of the abdomen. 4. The non-appearance, subsequently, of hernia, although the patient has never worn a bandage, is calculated to inspire doubts as to the danger of hernia, which, according to some surgeons, is much to be feared after wounds dividing the entire thickness of the abdominal parietes.

M. Lépine judiciously remarks that a more favorable opportunity of appreciating the part which the stomach performs in vomiting could not present itself. The stomach, filled with aliments recently ingested, escapes from the cavity of the abdomen, and the want to vomit manifests itself spontaneously. The phenomena, observed by M. Lépine, confirm the result of the experiments made by MM. Beclard and Magendie. During all the period that the stomach remained out of the abdominal cavity there was no apparent contraction of the muscular fibres of the organ, and none of its contents were expelled, although the patient made violent efforts to vomit. As soon, however, as the stomach had been returned into the abdomen, the same efforts were followed by the expulsion of its contents. This fact, as M. Lépine observed, shows that if the stomach be not entirely passive during the act of vomiting, at all events, the most important part is performed by the diaphragm and the abdominal muscles.

M. Lépine was also able to observe a phenomenon noticed by M. Magendie in his experiments on animals, and which he supposed likewise to exist in man. It seems that while vomiting, animals swallow a considerable quantity of air. Speaking of the enormous dilatation of the stomach by gases, M. Lépine remarks: I can only explain this distention by the air which the patient appeared to swallow after each effort of vomiting; I then observed him to perform repeatedly the act of deglutition, each deglutition being accompanied by a noise which seemed to be created by the pushing back of air.

NECROSIS OF A PORTION OF THE FRONTAL AND PARIETAL BONES.—PURULENT COLLECTION IN THE RIGHT HEMISPHERE OF THE BRAIN.—HEMIPLEGIA ON THE RIGHT SIDE.

This case presents great interest with reference to the relation which existed between the seat of the lesion of the brain and the parts of the body which were struck with paralysis. A man receives a blow on the right temporal region; several months afterwards he is seized with cephalalgia, and presents a puffy state of the integuments of the right temporal region, of the right cheek, and right ear. The functions of the brain become disturbed, and paralysis attacks the right arm and leg. An abscess forms on a level with the temporo-maxillary articulation; is opened, and the patient experiences temporary relief. Very soon, however, he falls into a still more serious state of prostration. His intellectual faculties entirely give way, the paralysis becomes complete on the right side, and he succumbs two months after the first apparition of the morbid symptoms.

The following are the lesions revealed by *the autopsy*:—Perforation of the bones of the skull at the level of the anterior and inferior angle of the right parietal. The internal lamella of the frontal and parietal is separated from the dura mater for an extent of two square inches, and is necrosed and perforated in several places; the dura mater is healthy, except a little redness and increased thickness opposite the lesion of the bones. The consistency of the brain is natural; its surface is not diseased in the region corresponding to the alteration of the bones of the skull. "The *right* hemisphere contained a considerable collection of healthy thick pus, which escaped as soon as the instrument had penetrated the cavity in which it was contained."

This last sentence is literally reproduced from Lépine's account of the case. We are sorry that he does not enter into greater details, as it is of great importance to know in what part of the hemisphere the abscess was situated, whether it was in the anterior, middle, or posterior lobe. Whatever may have been its situation, it is evident that the lesion of the brain was on the right side, and that the paralysis attacked the arm and leg on the same side. M. Lépine, in mentioning this circumstance, remarkes how unusual it is. All pathologists have, indeed, noticed the opposition which exists between the side of the head which is wounded and the side of the body which is paralysed. Hippocrates himself states that those who are wounded on the right side become impotent on the left, and *vice versa*. The authors who followed, although they recognised the correctness of this remark in a general sense, having sometimes observed the contrary, erroneously concluded that the side of the paralysis is not in relation with the local lesion of the brain. This error was owing to the situation of the external lesion being the one taken into consideration and compared to the paralysed side; whereas the seat of the internal cerebral lesion ought to have been the one considered. Nevertheless, cases which we owe to Morgagni and Lancisi, as also several observed in our own times by MM. Bayle and Dechambre, would seem to indicate that the paralysis may occupy the same side as the organic alteration. We must not forget, however, to recall to mind that these were cases of softening of the brain, and not of traumatic effusion. We know only one case, that of M. Blandin, in which the paralysis was on the same side as a traumatic effusion. Henceforth we shall have to add to this case the one narrated above to the French Academy. How can we explain facts so exceptional as these? Can we admit the explanation given by Gall and Spurzheim, and say, with them, that the presence of the cerebral lesion and of the paralysis on the same side is owing to the absence of decussation of olivary fasciculi of the spinal cord? This question requires elucidating.—*Lond. Lancet*, *Jan.* 20.

